# Association of Plasma Transferrin With Cognitive Decline in Patients With Mild Cognitive Impairment and Alzheimer’s Disease

**DOI:** 10.3389/fnagi.2020.00038

**Published:** 2020-03-12

**Authors:** Jingjing Guan, Peng Wang, Liping Lu, Guanan Zhao

**Affiliations:** ^1^Department of Pharmacy, Lishui City People’s Hospital, Lishui, China; ^2^Department of Clinical Laboratory, Lishui City People’s Hospital, Lishui, China; ^3^Department of Neurology, Lishui City People’s Hospital, Lishui, China; ^4^Department of General Surgery, Lishui City People’s Hospital, Lishui, China

**Keywords:** transferrin, iron, Alzheimer’s disease, mild cognitive impairment, longitudinal study

## Abstract

**Objective**: The objective of this study was to examine whether plasma transferrin levels are associated with longitudinal changes in cognitive performance in older individuals with normal cognition (CN), mild cognitive impairment (MCI), and mild Alzheimer’s disease (AD).

**Methods**: At baseline, there were a total of 358 participants from the Alzheimer’s Disease Neuroimaging Initiative (ADNI) cohort, including 58 older individuals with CN, 198 older individuals with MCI, and 102 patients with AD. Linear mixed models were utilized to examine the associations of plasma transferrin levels with changes in cognitive performance over time after adjustment of several potential covariates. The Mini-Mental State Examination (MMSE) and the Clinical Dementia Rating Scale Sum of Boxes (CDR-SB) were used to examine the global cognition of participants.

**Results**: First, no significant differences in the plasma transferrin levels were observed across three diagnostic groups. Second, in the cross-sectional analyses, the baseline plasma transferrin levels were negatively associated with the MMSE scores in the CN group, but not in the MCI or the AD group. Third, in the longitudinal analyses, we found that a higher plasma transferrin was associated with a steeper cognitive decline in the MCI and AD groups, but not in the CN group.

**Conclusion**: Higher plasma transferrin levels were associated with a steeper cognitive decline in participants with MCI and AD.

## Introduction

A growing body of evidence has suggested that iron elevation plays a crucial role in the pathogenesis of Alzheimer’s disease (AD; Lane et al., 2018; Nikseresht et al., 2019). It has been reported that brain iron may combine with amyloid-β (Aβ) to propel cognitive decline (Ayton et al., 2017b, 2019). Cerebrospinal fluid (CSF) ferritin levels were found to be associated with longitudinal changes in cognition, brain glucose metabolism, and CSF Aβ42 (Ayton et al., 2015, 2018; Diouf et al., 2019). A clinical trial of 48 patients with AD suggests that deferoxamine, an iron chelator, may slow the clinical progression (Crapper McLachlan et al., 1991). While evidence in both preclinical and clinical studies support the notion that brain iron elevation could contribute to cognitive decline and the incidence of dementia (Lane et al., 2018; Nikseresht et al., 2019), prospective evidence about the association between plasma transferrin (which transports iron) and changes in cognitive performance over time is lacking.

Transferrin is a crucial circulating protein that is involved in the mobilization and transformation of iron (Kawabata, 2019). It has been reported that the transferrin C2 allele is associated with a higher risk of developing AD dementia (Namekata et al., 1997; Zambenedetti et al., 2003). Previous cross-sectional studies did not observe a significant difference in blood transferrin levels between healthy controls and AD patients (Fischer et al., 1997; Squitti et al., 2010; Torsdottir et al., 2011). Blood transferrin levels were reported to be positively associated with the Mini-Mental State Examination (MMSE) scores in AD patients (Fischer et al., 1997), but this association was not replicated in another study (Squitti et al., 2010). Moreover, no study has attempted to examine the association between baseline plasma transferrin levels and changes in cognitive performance over time.

In this study, we compared the levels of transferrin in plasma among individuals with normal cognition (NC), individuals with mild cognitive impairment (MCI), and patients with mild AD dementia from the Alzheimer’s Disease Neuroimaging Initiative (ADNI) cohort. Second, the cross-sectional relationships between plasma transferrin and cognitive performance were examined across three diagnostic groups. Third, a linear mixed model was utilized to examine the association of plasma transferrin and changes in cognition over time.

## Materials and Methods

### Alzheimer’s Disease Neuroimaging Initiative

The present study used data downloaded from the ADNI on 12 July 2019 (adni.loni.usc.edu). The ADNI Study has been previously described in detail (Weiner et al., 2015). The primary aim of the ADNI Study has been to assess whether an array of markers, such as neuropsychological assessments, fluid biomarkers, and neuroimaging markers, can be integrated to predict the progression of MCI and AD dementia. At each participating ADNI center, the institutional review board approved the ADNI Study. All participants provided written informed consent.

### Participants

In this study, participants who had baseline plasma transferrin data and follow-up evaluation of global cognition were included. At baseline, there were a total of 358 participants, including 58 participants with NC, 198 participants with MCI, and 102 participants with mild AD dementia. The criteria for these three diagnostic groups can be found in the ADNI website^1^. The numbers of participants at baseline and at each follow-up visit are listed in Table 1.

**Table 1:** Demographics and clinical variables.

Variables	NC (*n* = 58)	MCI (*n* = 198)	AD (*n* = 102)	*p*-value
Age (years)	75.1 ± 5.77	74.3 ± 7.48	74.9 ± 7.91	0.71
Education (years)	15.7 ± 2.78	15.8 ± 2.99	15.1 ± 3.29	0.18
Female gender, *n* (%)	28 (48.3)	65 (32.8)	43 (42.2)	0.06
APOE4, *n* (%)	5 (8.6)	106 (53.5)^a^	71 (69.6)^b,c^	<0.001
MMSE scores	28.9 ± 1.15	26.9 ± 1.79^a^	23.5 ± 1.89^b,c^	<0.001
CDR-SB scores	0.03 ± 0.11	1.56 ± 0.86^a^	4.27 ± 1.56^b,c^	<0.001
Plasma transferrin (mg/dl)	3.42 ± 0.09	3.44 ± 0.07	3.44 ± 0.08	0.26
Tau/Aβ42 ratio	0.26 ± 0.09	0.74 ± 0.59^a^	0.9 ± 0.46^b,c^	<0.001
Subjects present at baseline and at each follow-up evaluation, *n*
Baseline	58	198	102
1 year	57	185	91
2 years	52	160	80
2 years	51	132	–	
2 years	34	66	–	
5 years	28	54	–	
6 years	30	51	–	

*Abbreviations: NC, normal cognition; MCI, mild cognitive impairment; AD, Alzheimer’s disease; MMSE, Mini-Mental State Examination; CDR-SB, Clinical Dementia Rating Scale Sum of Boxes. Notes: ^a^NC vs. MCI. ^b^NC vs. AD. ^c^MCI vs. AD*.

### Cognition

The MMSE (Folstein et al., 1975) was used to examine the global cognition of each participant. The MMSE test scores range from 0 to 30, with lower scores indicating a more severe degree of cognitive impairment. The MMSE was administrated at baseline and at each follow-up visit. In addition, the Clinical Dementia Rating Scale Sum of Boxes (CDR-SB; Morris, 1993) was also utilized to examine the global cognition of participants. A higher CDR-SB score indicates a more severe degree of cognitive deficits.

### Measurement of Plasma Transferrin

Baseline plasma transferrin was determined by the Biomarker Consortium Project Team using a multiplex immunoassay panel, which was developed on the Luminex xMAP platform by Rules-Based Medicine (RBM). The analyte was log-transformed to better approximate normality. The value was given as milligrams per deciliter. In the linear mixed models, the plasma transferrin levels were categorized into the high group and the low group based on the median (3.434 mg/dl).

### Statistical Analyses

ANOVA models and *χ*^2^ tests were used to assess differences in the demographics and clinical variables across the three diagnostic groups. To examine the cross-sectional relationships between the plasma transferrin levels and MMSE scores, Spearman’s correlation tests were utilized in the whole sample and within each diagnostic group. Further, to examine the associations of the baseline plasma transferrin levels with changes in MMSE and CDR-SB over time, linear mixed models were performed for the whole sample. To examine whether the clinical diagnosis modifies the associations of plasma transferrin with changes in MMSE and CDR-SB, linear mixed models were performed within each diagnostic group separately. Models were adjusted for age, gender, educational levels, APOE4 genotype, and tau/Aβ ratio. All linear mixed models included a random intercept for each subject. There were severe drop-offs in individuals after 2 years in the AD group and ~6 years for the CN and MCI groups. Therefore, the data in the model have been limited to ~6 years for the CN and MCI groups and ~2 years for the AD group. The statistical work was conducted with R software (version 3.6.0).

## Results

### Demographic and Clinical Variables

At baseline, there were a total of 358 participants, including 58 participants with NC, 198 participants with MCI, and 102 participants with mild AD dementia (Table 1). As shown in Table 1, there were significant differences in the percentage of APOE4 carriers, MMSE scores, CDR-SB scores, and the tau/Aβ42 ratio across the three diagnostic groups (all *p* < 0.001). However, no significant differences in the other demographics (age, educational years, or gender) were observed (all *p* > 0.05). Further, no significant differences in the plasma transferrin levels were found across the three diagnostic groups. The numbers of participants at baseline and at follow-up visits are also listed in Table 1.

### Cross-Sectional Relationships Between Plasma Transferrin and Cognition in the Three Diagnostic Groups

**Figure 1:**
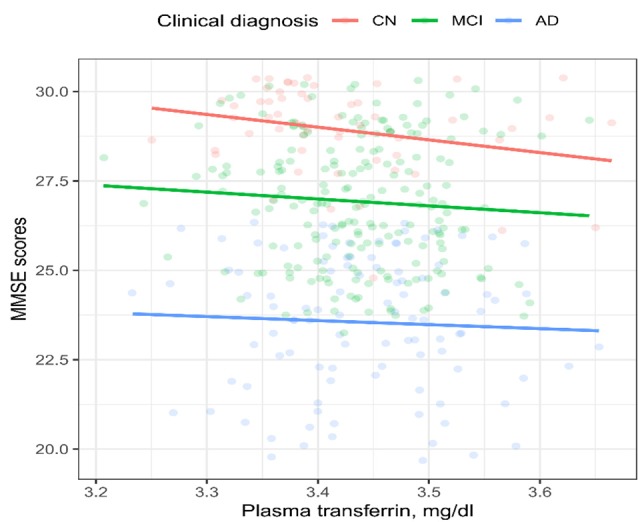
Cross-sectional relationship between the plasma transferrin levels and the MMSE scores in the three diagnostic groups. The plasma transferrin levels were negatively associated with the MMSE scores in the NC group (*ρ* = −0.27, *p* = 0.036), but not in the MCI group (*ρ* = −0.1, *p* = 0.33) or in the AD group (*ρ* = −0.04, *p* = 0.7). NC, normal cognition; MCI, mild cognitive impairment; AD, Alzheimer’s disease; MMSE, mini-mental state examination.

To examine the cross-sectional relationships between the plasma transferrin levels and MMSE scores, Spearman’s correlation tests were performed within each diagnostic group (Figure 1). We found that the plasma transferrin levels were negatively associated with the MMSE scores in the NC group (*ρ* = −0.27, *p* = 0.036), but not in the MCI group (*ρ* = −0.1, *p* = 0.33) or in the AD group (*ρ* = −0.04, *p* = 0.7).

### Longitudinal Associations of Baseline Plasma Transferrin With Cognition in the Three Diagnostic Groups

**Figure 2:**
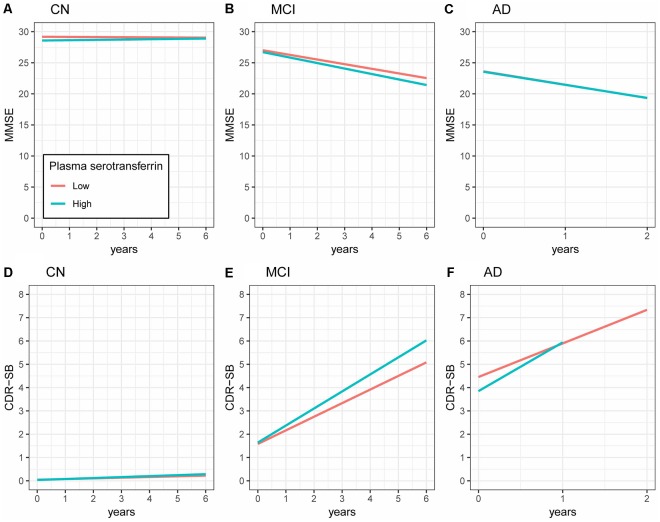
Longitudinal associations of plasma transferrin at baseline with the MMSE and CDR-SB scores within each diagnostic group. **(A–C)** Higher plasma transferrin levels were not associated with changes in the MMSE scores in the CN, MCI, or the AD group (all *p* > 0.05). **(D–F)** Higher plasma transferrin levels were associated with a steeper increase in the CDR-SB scores among patients with MCI (estimate = 0.15, *p* = 0.009) and AD (estimate = 0.59, *p* = 0.007), but not the CN group (estimate = 0.01, *p* = 0.45). NC, normal cognition; MCI, mild cognitive impairment; AD, Alzheimer’s disease; MMSE, Mini-Mental State Examination ; CDR-SB, Clinical Dementia Rating Scale Sum of Boxes.

To examine the longitudinal associations of the plasma transferrin levels at baseline with the MMSE scores, linear mixed models were performed within each diagnostic group. As shown in Table 2 and Figure 2, higher plasma transferrin levels were not associated with changes in the MMSE scores in the CN, MCI, or the AD group (all *p* > 0.05).

**Table 2:** Summary of the linear mixed models.

	MMSE		CSR-SB	
	Estimate (SE)	*p*-value	Estimate (SE)	*p*-value
In the NC group
Transferrin (high vs. low) × time	0.07 (0.06)	0.24	0.01 (0.02)	0.45
In the MCI group
Transferrin (high vs. low) × time	−0.13 (0.09)	0.15	0.15 (0.06)	0.009
In the AD group
Transferrin (high vs. low) × time	−0.01 (0.4)	0.98	0.59 (0.22)	0.007

*All models were adjusted for age, gender, educational years, APOE4 genotype, and tau/Aβ42. The estimates represent the amount of changes in the MMSE scores per year. NC, normal cognition; MCI, mild cognitive impairment; AD, Alzheimer’s disease; MMSE, Mini-Mental State Examination; CDR-SB, Clinical Dementia Rating Scale Sum of Boxes*.

To examine the longitudinal associations of the plasma transferrin levels at baseline with the CDR-SB scores, linear mixed models were performed within each diagnostic group. As shown in Table 2 and Figure 2, higher plasma transferrin levels were associated with a steeper increase in the CDR-SB scores among patients with MCI (estimate = 0.15, *p* = 0.009) and AD (estimate = 0.59, *p* = 0.007), but not the CN group (estimate = 0.01, *p* = 0.45).

We further classified the MCI subjects into three groups based on the tertiles of plasma transferrin. Several linear mixed models were used to compare the slopes of cognitive decline between the three tertiles among the MCI subjects (please see Supplementary Data Sheet 1).

## Discussion

There were several findings in the present study. First, no significant differences in the plasma transferrin levels were observed across the three diagnostic groups. Second, in the cross-sectional study, the baseline plasma transferrin levels were negatively associated with the MMSE scores in the NC group, but not in the MCI or the AD group. Finally, in the longitudinal study, we found that a higher plasma transferrin was associated with a steeper cognitive decline in the MCI and AD groups, but not in the NC group.

In the cross-sectional study, we found that plasma transferrin was negatively associated with the MMSE scores in older individuals with NC, but not in MCI or AD patients. The negative association between plasma transferrin and the MMSE scores observed in individuals with NC may indicate that higher levels of plasma transferrin could have adverse effects on cognitive performance among cognitively normal older people. However, Fischer et al. (1997) found that serum levels of transferrin were positively associated with the MMSE scores in 41 AD patients (Fischer et al., 1997), which is not in line with our finding in AD patients. This inconsistency may be due to several potential contributors, such as: (1) the different sample, which could be serum or plasma; (2) the sample size; (3) different severities of dementia; and (4) the different methodological techniques used to determine the levels of transferrin.

In the longitudinal study, the finding that higher plasma transferrin levels at baseline were associated with a steeper cognitive decline in older individuals with MCI and AD is novel. Regarding the biology of transferrin, it plays a crucial role in the mobilization and transformation of iron (Kawabata, 2019). Human transferrin is a 76-kDa protein that is primarily expressed in the liver and has a half-life of 8 days (Kemp et al., 1984). A large portion of brain iron originates from the transferrin iron in the blood (Moos et al., 2006, 2007; Skjørringe et al., 2015). It has been suggested that brain iron accumulation is a key feature of AD (van Rooden et al., 2015) and might play an important role in the oxidative damage found in AD brains (Smith et al., 1997). Ferritin is another iron-binding protein, and higher CSF ferritin levels were reported to be associated with a greater cognitive decline over a period of 7 years among cognitively normal older individuals carrying the APOE4 allele (Ayton et al., 2017a). Further, a clinical trial of 48 AD patients suggested that deferoxamine, an iron chelator, may slow the clinical progression (Crapper McLachlan et al., 1991). Collectively, these data strengthen the notion that the dysregulation of iron metabolism might contribute to the pathogenesis of AD, and lowering the levels of plasma transferrin should be considered as a therapeutic strategy to slow the cognitive decline in MCI and AD patients. However, this notion should be tested in further clinical trials.

There are several limitations in the present study. First, in the longitudinal study, we did not observe an association between plasma transferrin and cognitive decline in the NC group. This may be due to the fact that the number of participants in the NC group was relatively smaller than that in the MCI group. Thus, the association between the plasma transferrin levels and cognitive decline in the NC group will need further investigation. Second, participants recruited in the present study represent a convenience sample, and this may limit the generalization of our findings. Third, the causal association between plasma transferrin and cognitive decline cannot be determined due to the study design. Further clinical trials will be warranted. Finally, we cannot rule out the possibility that other analytes may have some potential effects on the levels of plasma transferrin due to the fact that transferrin was measured by a multiplex assay rather than a more targeted assay. Therefore, our results should be further validated in a future study with a more targeted assay.

In conclusion, higher plasma transferrin levels were associated with a steeper cognitive decline in patients with MCI and AD.

## Data Availability Statement

The datasets generated for this study will not be made publicly available. The datasets are available at the ADNI website (http://adni.loni.usc.edu).

## Ethics Statement

The studies involving human participants were reviewed and approved by the ADNI centers across the USA and Canada. The patients/participants provided their written informed consent to participate in this study.

## Author Contributions

GZ conceived and designed the study. JG, PW, and LL analyzed and interpreted the data. JG drafted and revised the manuscript. All authors revised and approved the final version of the manuscript.

## Conflict of Interest

The authors declare that the research was conducted in the absence of any commercial or financial relationships that could be construed as a potential conflict of interest.

## Footnotes

^1^http://adni.loni.usc.edu/wp-content/uploads/2010/09/ADNI_GeneralProceduresManual.pdf
