# Supplementary material for: Association of Plasma Transferrin With Cognitive Decline in Patients With Mild Cognitive Impairment and Alzheimer’s Disease
Source: Front Aging Neurosci. 2020 Mar 12;12:38. doi: 10.3389/fnagi.2020.00038 (PMC7080847; doi:10.3389/fnagi.2020.00038)
Supplement: Supplementary file 1 [file Data_Sheet_1.pdf]

Supplementary Material

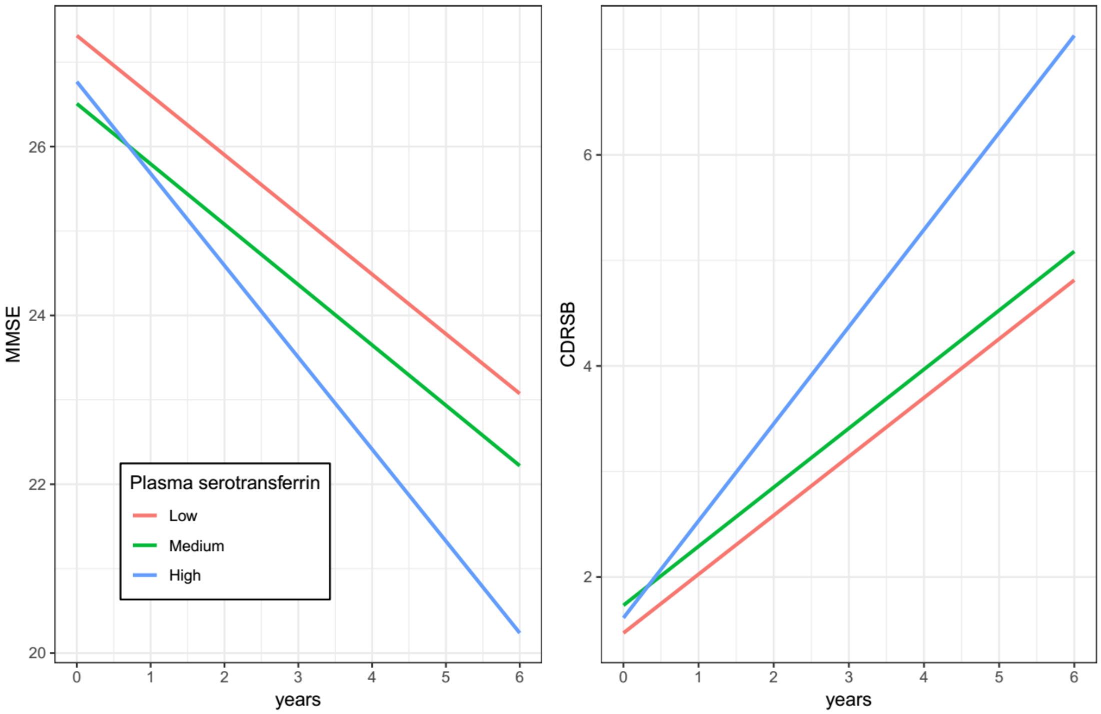

Supplementary Figure. Association of plasma transferrin with cognitive decline among MCI subjects. Compared to individuals in the low group, those in the high group had a steeper decline in cognitive performance over a period of 6 years (MMSE: estimate: -0.38, $p = 0.001$; CDRSB: estimate: 0.36, $p < 0.001$). However, no significant difference in slopes of cognitive decline (MMSE or CDRSB) was observed between the low group and the medium group (all $p > 0.05$).

Supplementary Table. Association of plasma transferrin with cognitive decline among MCI subjects.

	MMSE		CSR-SB	
	Estimate (SE)	P value	Estimate (SE)	P value
Transferrin (Medium vs Low) × time	-0.01 (0.1)	0.95	0.00 (0.07)	1
Transferrin (High vs Low) × time	-0.38 (0.12)	0.001	0.36 (0.07)	< 0.001

Abbreviations: NC: normal cognition; MCI: mild cognitive impairment; AD: Alzheimer's disease, MMSE: mini-mental state examination. CDR-SB: Clinical dementia rating -sum of boxes. Notes: All models were adjusted for age, gender, educational years, APOE4 genotype and tau/A β 42.
